# Prognosis of ampullary cancer based on immunohistochemical type and expression of osteopontin

**DOI:** 10.1186/1746-1596-6-98

**Published:** 2011-10-13

**Authors:** Xiang-qian Zhao, Jia-hong Dong, Wen-zhi Zhang, Zhe Liu

**Affiliations:** 1 Hospital & Institute of Hepatobiliary Surgery, Chinese PLA General Hospital, 28 Fuxing Road, Beijing 100853, China

**Keywords:** ampullary cancer, osteopontin, survival analysis, immunohistochemistry, classification, Cytokeratin 20, Cytokeratin 7

## Abstract

**Background:**

Ampullary cancer (AC) was classified as pancreatobiliary, intestinal, or other subtype based on the expression of cytokeratin 7 (CK7) and cytokeratin 20 (CK20). We aimed to explore the association of AC subtype with patient prognosis.

**Methods:**

The relationship of AC subtype and expression of Osteopontin (OPN) with the prognosis of 120 AC patients after pancreaticoduodenectomy was investigated.

**Results:**

The patients had pancreatobiliary (CK7^+^/CK20^-^, n = 24, 20%), intestinal (CK7^-^/CK20^+^, n = 29, 24.2%) or other (CK7^+^/CK20^+ ^or CK7^-^/CK20^-^, n = 67, 55.8%) subtypes of AC, and their median survival times were 23 ± 4.2, 38 ± 2.8 and 64 ± 16.8 months, respectively. The survival times of 64 OPN^- ^patients (53.3%) and 56 OPN^+ ^patients (46.7%) were 69 ± 18.4 and 36 ± 1.3 months, respectively. There was no significant effect of AC subtype on survival of OPN^- ^patients. For OPN^+ ^patients, those with pancreatobiliary AC had a shorter survival time (22 ± 6.6 months) than those with intestinal AC (37 ± 1.4 months, *p *= 0.041), and other AC subtype (36 ± 0.9 months, *p *= 0.010); intestinal and other AC subtypes had similar survival times.

**Conclusions:**

The prognosis of AC patients can be estimated based on immunohistochemical classification and OPN status.

## Background

Ampullary carcinoma (AC) is a relatively rare tumor of the hepatopancreatic ampulla that accounts for approximately 0.2% of gastrointestinal tract malignancies and 7% of periampullary carcinomas [1]. ACs have different anatomical origins. Kimura et al. initially classified AC as pancreatobiliary AC if it had papillary projections with scant fibrous cores and as intestinal AC if it resembled tubular adenocarcinoma of the stomach or colon [2]. Numerous studies have reported that intestinal AC is associated with a better prognosis than pancreatobiliary AC [1-5].

AC has also been classified based on immunohistochemical expression of cytokeratin 7 (CK7), Mucins and CDX2 [4,6-8] and HNF4α [9]. However, the clinical significance and survival rates of AC patients with these different immunohistochemical subtypes have not been definitely established.

Histologic classification and immunohistochemical characterization by cytokeratins are in good agreement [5]. Fischer et al. reported that the histological subtypes of AC could be determined by the expression of CK7, CK20, and MUC2; pancreaticobiliary AC is CK7^+^/CK20^-^/MUC2^-^, and intestinal AC is CK7^-^/CK20^+^/MUC2^+ ^[10]. Zhou et al. classified CK7^-^/CK20^+ ^tumors as intestinal AC, CK7^+^/CK20^- ^tumors as pancreatobiliary AC, and tumors that are CK7^+^/CK20^+ ^or CK7^-^/CK20^- ^as "other" [3]. However, there was no statistical difference in survival of patients with different CK7/CK20 subtypes [3] or with different CK20/MUC subtypes [11].

Osteopontin (OPN) is a secretory calcium-binding phosphorylated glycoprotein and plays an important role in bone metabolism. OPN is widely distributed in the urine, blood, gastrointestinal tract, pancreas, lungs and elsewhere. At the molecular level, OPN plays important roles in cellular adhesion and migration, tissue repair, and signal transduction and also in the invasion and metastasis of several cancers [12]. OPN is significantly associated with survival rate in several cancers and has value as a marker of clinical tumor progression [13,14]. In particular, low OPN levels were significantly associated with a favorable prognosis in patients with advanced non-small cell lung cancer [15], laryngeal and hypopharyngeal carcinomas [16], hepatocellular carcinoma [17], colorectal cancer [18], idiopathic pulmonary hypertension [19], upper urinary tract urothelial carcinoma [20], acute myeloid leukemia [21], oral squamous cell carcinoma [22], and endometrial cancer [23]. OPN may also be a suitable biomarker for overall survival and renal outcome of patients who are critically ill with acute kidney injury [24].

However, few studies have investigated the expression of OPN in patients with AC. Van Heek et al. reported higher OPN expression in the sera and tumors of AC patients than in the sera and duodenal samples of healthy controls [25]. Bloomston et al. reported that node-negative status and lack of OPN expression were associated with prolonged survival in patients with AC [26]. Hsu et al. reported that expression of OPN and the presence of tumor-associated macrophages in bulky AC were associated with tumor recurrence, and poorer disease-specific survival [27].

In the present study, we retrospectively analyzed the clinical data of 120 patients who were undergoing pancreaticoduodenectomy due to AC. We focused on the association of AC prognosis with the expression of CK7, CK20, and OPN.

## Patients and Methods

### Patients

From January 1, 1994 to December 30, 2008, patients undergoing pancreaticoduodenectomy due to AC were recruited from the Department of Hepatobiliary Surgery of the General Hospital of the Peoples Liberation Army (Beijing, China). The exclusion criteria were: ***(i) ***duodenal cancer, cancer of the lower bile duct, or cancer of the pancreas or any of these cancers involving the ampulla or duodenal papilla, based on pathological examination; ***(ii) ***uncertain origin of the cancer; ***(iii) ***previous focal resection of duodenal papillary cancer or AC; ***(iv) ***metastasis to other organs; and ***(v) ***presence of concomitant heart disease, cerebrovascular disease, or pulmonary disease that made the patient ineligible for surgery. Follow-up examinations were performed at 3 months after surgery, once every 6 months for 3 years, and then once per year. These follow-up examinations included routine tests (liver and kidney function, blood electrolytes, routine blood test), tests for tumor markers, chest X-ray, and abdominal imaging by ultrasonography, CT, or MRI. The last follow-up was on January 31, 2010. Tumor stage and lymph node metastasis were evaluated according to Greene et al [28]. This study was approved by the hospital Institutional Review Board.

### Immunohistochemistry

Carcinoma specimens were embedded in paraffin, cut consecutively into sections (4 μm), and the streptavidin-peroxidase method was used for immunohistochemical visualization (UltraSensitive™ SP kit, Maximbio. Co. Ltd, Fuzhou, China). The primary antibodies were mouse anti-human CK7 or CK20 monoclonal antibodies and rabbit anti-human OPN polyclonal antibody (Lab Vision & NeoMarkers, USA). The normal serum from non-immunized goat was used as a negative control of the primary antibody, and CK7^+^/CK20^+^/OPN^+ ^pancreatic carcinoma was used as a positive control.

Details for the determination of positive staining were previously provided[3]. In brief, cells positive for CK7, CK20, or OPN had brown or yellow-brown granules, mainly in the cytoplasm. Sections were evaluated by two independent and blinded pathologists. No staining or staining in fewer than 10% of cells was considered negative, and staining of 10% or more of cells was considered positive.

### Statistical analysis

Results are expressed as means or medians with standard deviations, or counts and percentages. Survival analysis was analyzed by the Kaplan-Meier method and the log-rank test. Data were analyzed using SPSS 15.0 (SPSS, Inc., Chicago, IL, USA). All *p*-values were two-sided and were considered significant if *p *was less than 0.05.

## Results

### Patient characteristics

A total of 120 patients (84 males, 36 females) met our inclusion criteria and received follow-up examinations. The mean age was 55.1 ± 9.8 years and the mean tumor diameter was 2.4 ± 1.5 cm. The 1-, 3- and 5-year survival rates were 94.8%, 78.7%, and 68.0%, respectively. The median survival time was 38 ± 11.3 months and the mean survival time was 53.9 months. A total of 51 patients (42.5%) survived to the end of the follow-up period (January 31, 2010), 1 patient survived more than 10 years, and 2 patients survived more than 5 years. In addition to pancreaticoduodenectomy (given to all patients), 8 patients received chemotherapy. Among the 69 patients (57.5%) who died in the follow-up period, 21 died within 5 years after surgery and 1 died 11 years after surgery.

### Survival of patients with different subtypes of AC

**Figure 1:**
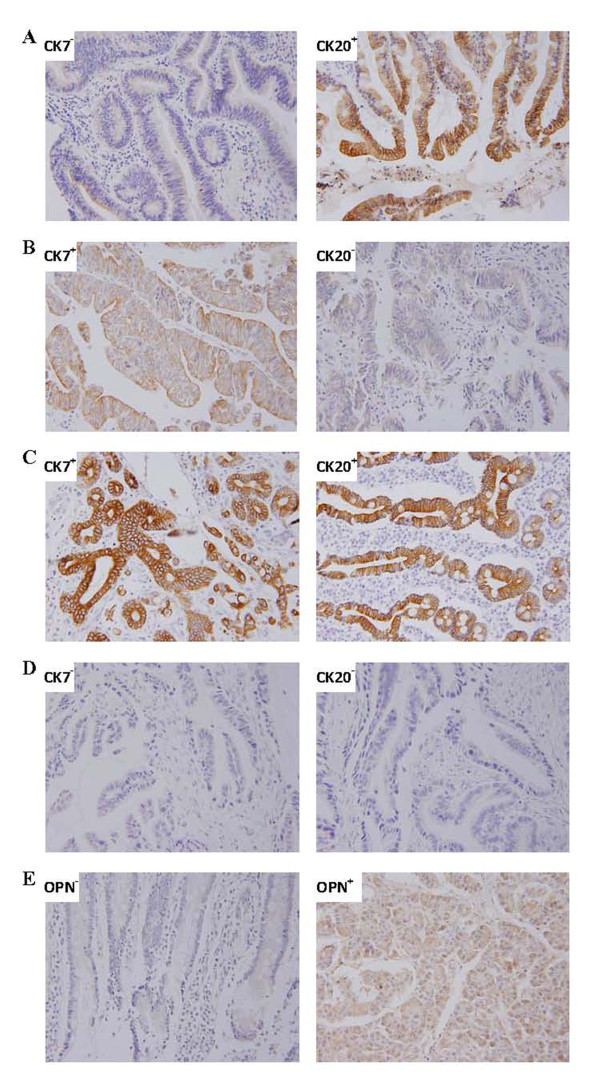
**Representative immunohistochemical staining of ampullary cancer**. A: intestinal type, CK7^-^/CK20^+^; B: pancreatobiliary type, CK7^+^/CK20^-^; C: other type, (C: CK7^+^/CK20^+^); D: other type, CK7^-^/CK20^-^); E: positive and negative immunohistochemical staining for osteopontin.

Figure 1A-D shows representative immunohistochemical results of patients with intestinal AC (CK7^-^/CK20^+^), pancreatobiliary AC (CK7^+^/CK20^-^), and other subtype of AC (CK7^+^/CK20^+ ^and CK7^-^/CK20^-^). Figure 1E shows representative positive and negative results for OPN staining.

**Figure 2:**
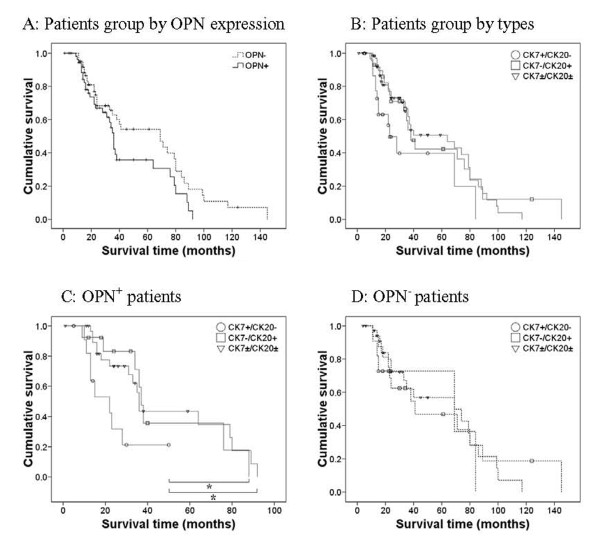
**Kaplan-Meier survival curves of patients with different immunohistochemical types of ampullary cancer and with positive or negative expression of osteopontin**. *p < 0.05 by log-rank test.

Table 1 shows the survival times of AC patients stratified by immunohistochemical results, extent of tumor differentiation, amount of tumor invasion, and lymph node metastasis. None of the other associations were significant. OPN^+ ^patients had marginally longer survival time than OPN^- ^patients (*p *= 0.062; Figure 2A). The 1-, 3, and 5-year survival rates were 84.3%, 55.3%, and 0% for OPN^+ ^patients and 91.8%, 68.4% and 7.2% for OPN^- ^patients respectively.

**Table 1:** Characteristics of ampullary carcinoma and survival time of patients.

Variable	N (%)	Survival (months)	time P value
**Immunohistochemical Staining**			
CK7^-^	40 (33.3)	41.0 ± 20.0	0.166
CK7^+^	80 (66.7)	36.0 ±3.7	
CK20^-^	35 (39.2)	31.0 ± 24.3	0.316
CK20^+^	85 (70.8)	38.0 ± 3.2	
OPN^-^	64 (53.3)	69.0 ± 18.4	0.062
OPN^+^	56 (46.7)	36.0 ± 1.3	
Total	120 (100)	38 ± 11.3	
**Tumor Differentiation**			0.408
Low	34 (28.3)	35.0 ± 3.4	
Medium	37 (30.8)	36.0 ± 15.7	
High	49 (40.8)	41.0 ± 18.9	
**Tumor Invasion**			0.332
T1	57 (47.5)	71.0 ± 23.0	
T2	16 (13.3)	69.0 ± 0.0	
T3	47 (39.2)	35.0 ± 5.9	
**Lymph node metastasis**			0.275
No	88 (73.3)	38.0 ± 3.6	
Yes	32 (26.7)	40.0 ± 24.8	

Survival time is expressed as median ± SD and *p*-values were calculated by the log-rank test.

Figure 2B shows the survival times of patients with pancreatobiliary AC, intestinal AC, and other AC. Although pancreatobiliary AC was associated with slightly worse prognosis, a log-rank test indicated that this difference was only marginal (*p *= 0.052, Table 2). Figures 2C and 2D show the survival times of these three groups stratified by OPN expression. The results indicate that OPN^- ^patients with different subtypes of AC had similar survival times (*p *= 0.75, Table 2). However, OPN^+ ^patients with different subtypes of AC had significantly different survival times (*p *= 0.017, Table 2). Specifically, OPN^+ ^patients with pancreatobiliary AC had worse prognosis than those with intestinal AC (*p *= 0.041) and other subtype of AC (*p *= 0.010); the survival time of the patients with intestinal and other types had no difference. In addition, the survival time of patients with OPN^+ ^pancreatobiliary AC was shorter than those with OPN^- ^intestinal AC (*p *= 0.043), and other subtype of AC (*p *= 0.002).

**Table 2:** Expression of OPN and survival time of patients with different subtypes of ampullary carcinoma (AC).

Survival time in months (number of patients)				
	Pancreatobiliary	Intestinal	Other	p value^1^
Total	23 ± 4.2 (24)	38 ± 2.8 (29)	64 ± 16.8 (67)	0.052
OPN^-^	69 ± 39.5 (11)	41 ± 24.8 (16)	69 ± 26.8 (37)	0.750
OPN^+^	22 ± 6.6 (13)*	37 ± 1.4 (13)	36 ± 0.9 (30)	0.017
p value^2^	0.085	0.509	0.263	

Survival time is expressed as median ± SD. ^1^*p*-value for comparison of the 3 types; ^2^*p*-value for comparison of the OPN^+ ^and OPN^- ^groups. * *p *= 0.041 and *p *= 0.010 for comparison with the intestinal type and other type OPN^+ ^AC, respectively. There was no difference between intestinal AC and other type of AC (*p *= 0.907).

## Discussion

Many previous studies have examined the expression of histological markers in the carcinomas of patients with ACs. For example, de Paiva Haddad et al. reported that MUC1, MUC2, and CDX2 provided the best agreement with histomorphological classification of AC [4]. However, their multivariate analysis indicated that neither histological classification nor immunohistochemical results were statistically independent predictors of poor prognosis. Moriya et al. reported that MUC1 and MUC2 expression was useful for classification of pancreatobiliary and intestinal AC, and that pancreatobiliary AC was associated with worse prognosis than intestinal AC [8]. Ehehalt et al. reported that immunohistochemical determination of HNF4α expression distinguished different AC subtypes and that HNF4α protein expression was an independent predictor of favorable prognosis [9]. Westgaard et al. classified ACs by expression of CK7, MUC4, and CDX2 [6]. They found only moderate agreement of the immunohistochemical and histomorphological classifications, and that expression of MUC1 and/or MUC4 was independently associated with poor prognosis. Santini et al. reported that MUC2 and MUC5 expression was not associated with prognosis of the patients with radically resected AC [7]. Clearly, it is difficult to completely reconcile these diverse and occasionally contradictory results, which have examined many markers in diverse patient populations.

Other researchers have reported immunohistochemical classification of AC based on expression of CK7 and CK20 [3,10]. Thus, in the present study we classified our AC patients as having pancreatobiliary AC (CK7^+^/CK20^-^, 20%), intestinal AC (CK7^-^/CK20^+^, 24.2%), or other subtype of AC (CK7^+^/CK20^+ ^or CK7^-^/CK20^-^, 55.8%). Thus, the majority of our Chinese patients had other subtype of AC, in contrast to Kimura et al. [2], who studied Japanese patients, and Zhou et al. [3] who studied German patients and reported that pancreatobiliary AC was the most common subtype. This difference might be due to population differences and/or to the different reactivity thresholds used in the immunohistochemical classification [29]. Our survival analysis indicated no significant differences in survival among patients with the different subtypes of AC, consistent with the report of Zhou et al. [3]. Taken together, these findings suggest the immunohistologic subtype of AC alone has limited value in determination of the prognosis.

Previous research has indicated that OPN expression is significantly associated with poor survival of patients with several forms of cancer [13,14]. It has also been reported that elevated OPN expression in AC patients predicts poor disease-specific survival [25-27]. However, when we pooled all AC subtypes, we found that survival time of OPN^- ^and OPN^+ ^patients had no significant difference. This is consistent with the results of Matsuzaki et al., who reported that OPN expression in AC patients was not associated with survival rate, although OPN expression in the carcinoma was higher than in adjacent normal tissues [30].

Our further analysis indicated that the subtype of AC (intestinal, pancreatobiliary, or other) had no significant effect on survival of patients with OPN^- ^carcinomas. However, for patients with OPN^+ ^carcinomas, those with intestinal AC or other subtype of AC had significantly better survival than those with pancreatobiliary AC. Thus, OPN expression appears to affect the biological behavior of AC, and this effect depends on the anatomical origin of the tumor. These results indicate that determination of the prognosis of patients with AC should consider OPN expression.

Previous studies have reported interactions of OPN with other proteins. For example, in situ proximity ligation analysis indicated a molecular interaction of OPN and CD44 and that elevated expression of these proteins were associated with increased mitosis and significantly enhanced gastrointestinal stromal tumor cell proliferation in vitro [31]. Yang et al. reported that OPN combined with CD44 was a promising independent predictor of tumor recurrence and survival in patients with hepatocellular carcinoma [32]. OPN combined with CDX2 appears to predict survival of advanced gastric cancer patients, and CDX2 may be a transcription factor that modulates the expression of osteopontin [33]. Our results support previous reports which suggest that OPN has a role in the pathogenesis of AC [25-27]. However, a limitation of our study is that patients were enrolled retrospectively, and we did not include histomorphological classification of patients or immunochemical determination based on other factors including CDX2 and mucins. Clearly, the potential interaction of OPN, CK7, CDX2, mucins and other factors and the role of these in the pathogenesis of AC warrant further studies.

## Conclusions

In conclusion, our results indicate that it is difficult to determine prognosis of patients with AC based solely on immunohistochemical classification that considers CK7 and CK20 status. However, the additional consideration of OPN status allows determination of prognosis. Our results also suggest that OPN plays a role in the pathogenesis of AC, but its mechanisms and relationship with CK7 and CK20 warrant further studies.

## Abbreviations

AC: ampullary cancer; OPN: osteopontin; CK7: Cytokeratin 7; CK20: Cytokeratin 20.

## Completing interests

The authors declare that they have no competing interests.

## Authors' contributions

XQZ carried out the study design, defined the intellectual content, participated in the literature research and manuscript preparation, analyzed data, and edited the manuscript. JHD do guarantor of integrity of the entire study, carried out the study concepts, and reviewed the manuscript. WZZ carried out the clinical studies, and acquired data. ZL carried out the experimental studies, and did statistical analysis. All authors read and approved the final manuscript.
